# A CT-based radiomics model to detect prostate cancer lymph node metastases in PSMA radioguided surgery patients

**DOI:** 10.1007/s00259-020-04864-1

**Published:** 2020-05-28

**Authors:** Jan C. Peeken, Mohamed A. Shouman, Markus Kroenke, Isabel Rauscher, Tobias Maurer, Jürgen E. Gschwend, Matthias Eiber, Stephanie E. Combs

**Affiliations:** 1 Department of Radiation Oncology, Klinikum Rechts der Isar, School of Medicine, Technical University of Munich (TUM), Ismaninger Straße 22, 81675 Munich, Germany; 2 Institute of Radiation Medicine (IRM), Department of Radiation Sciences (DRS), Helmholtz Zentrum München, Neuherberg, Germany; 3 Deutsches Konsortium für Translationale Krebsforschung (DKTK), Deutsches Konsortium für Translationale Krebsforschung (DKTK), Partner Site Munich, Munich, Germany; 4 Department of Nuclear Medicine, Klinikum Rechts der Isar, School of Medicine, Technical University of Munich (TUM), Munich, Germany; 5 Institute for Diagnostic and Interventional Radiology, Klinikum Rechts der Isar, School of Medicine, Technical University of Munich (TUM), Munich, Germany; 6 Department of Urology, Klinikum Rechts der Isar, School of Medicine, Technical University of Munich (TUM), Munich, Germany; 7 Department of Urology and Martini-Klinik, University Hamburg-Eppendorf, Hamburg, Germany

**Keywords:** Radiomics, Prostate carcinoma, PSMA, CT, Lymph node, Radioguided surgery

## Abstract

**Purpose:**

In recurrent prostate carcinoma, determination of the site of recurrence is crucial to guide personalized therapy. In contrast to prostate-specific membrane antigen (PSMA)–positron emission tomography (PET) imaging, computed tomography (CT) has only limited capacity to detect lymph node metastases (LNM). We sought to develop a CT-based radiomic model to predict LNM status using a PSMA radioguided surgery (RGS) cohort with histological confirmation of all suspected lymph nodes (LNs).

**Methods:**

Eighty patients that received RGS for resection of PSMA PET/CT-positive LNMs were analyzed. Forty-seven patients (87 LNs) that received inhouse imaging were used as training cohort. Thirty-three patients (62 LNs) that received external imaging were used as testing cohort. As gold standard, histological confirmation was available for all LNs. After preprocessing, 156 radiomic features analyzing texture, shape, intensity, and local binary patterns (LBP) were extracted. The least absolute shrinkage and selection operator (radiomic models) and logistic regression (conventional parameters) were used for modeling.

**Results:**

Texture and shape features were largely correlated to LN volume. A combined radiomic model achieved the best predictive performance with a testing-AUC of 0.95. LBP features showed the highest contribution to model performance. This model significantly outperformed all conventional CT parameters including LN short diameter (AUC 0.84), LN volume (AUC 0.80), and an expert rating (AUC 0.67). In lymph node–specific decision curve analysis, there was a clinical net benefit above LN short diameter.

**Conclusion:**

The best radiomic model outperformed conventional measures for detection of LNM demonstrating an incremental value of radiomic features.

**Electronic supplementary material:**

The online version of this article (10.1007/s00259-020-04864-1) contains supplementary material, which is available to authorized users.

## Introduction

After initial therapy, biochemical failure in terms of a rising PSA level is the clinical evidence of a PC recurrence [1]. In this setting of locally recurrent prostate carcinoma (PC), patients regularly receive salvage radiotherapy (SRT) [2].

Besides the prostatic bed, pelvic lymph nodes (LNs) present a common site of recurrent disease that might alter clinical management. Using conventional imaging, it remains challenging to detect the exact site of recurrence to optimally guide personalized therapy [3]. Computed tomography (CT) is widely used for LN recurrence detection. In previous publications that used a short-axis diameter of pelvic LNs of 8 mm as an indicator for lymph node metastasis (LNM), only a limited sensitivity of 30–40% could be achieved [4, 5].

Previous studies could demonstrate inadequate coverage of recurrent disease using irradiation fields according to the Radiation Therapy Oncology Group (RTOG) clinical target volume (CTV) consensus guidelines [6, 7]. This may be explained by the tendency of PC to metastasize to uncommon sites after radical prostatectomy (RPE) and pelvic lymph node dissection (PLND) due to altered lymphatic drainage [8, 9].

^68^Ga-prostate-specific membrane antigen (PSMA)–positron emission tomography (PET) imaging has shown high accuracy in detecting LNMs. A recent meta-analysis found high sensitivities for LNM detection in recurrent PC ranging from 58 to 76% for accompanying PSA ranges of 0.2–1 ng/ml and 1–2 ng/ml [10]. A different study demonstrated the added value of using ^68^Ga-PSMA-11 PET/CT for SRT planning. Forty percent of all ^68^Ga-PSMA-11 positive LNs would not have been covered according to the treatment fields as defined by the RTOG consensus guidelines based on conventional imaging modalities [7].

PSMA radioguided surgery is a novel surgical approach that enables intraoperative detection and resection of PSMA PET-positive LNs following intravenous application of radioactively labeled PSMA with ^111^In-PSMA-I&T or ^99m^Tc-PSMA-I&S [11]. Maurer et al. could demonstrate that dissected LNs that showed a positive signal using a gamma-probe (radioactive rating) in vivo also harbored metastatic disease on histopathological evaluation [12]. In a recent analysis, ex vivo radioactive rating yielded a sensitivity of 83.6% and a specificity of 100% [13].

Radiomics describes the high-throughput extraction of quantitative features from medical imaging studies [14, 15]. Extracted features quantify intensity distributions, shape properties, and texture parameters such as “heterogeneity” in previously defined volumes of interest (VOI) [16, 17]. After incorporation into prediction models, such features can be used effectively to predict prognosis, histological properties, and molecular aberrations [18–21]. In PC, previous studies could demonstrate successful prediction of Gleason score and survival using radiomic models [22, 23].

To augment the evaluation of conventional CT for LN evaluation, we chose a radiomic approach to improve the diagnostic performance of CT for prediction of LNM in recurrent PC. We used a retrospective cohort of recurrent PC patients who underwent RGS due to ^68^Ga-PSMA-11 positive LNMs providing histological confirmation of all dissected LNs. Different radiomic feature sets were compared to find the optimal model. All models were validated using external imaging studies and compared to conventional CT parameters.

## Methods

### Patients

In this retrospective analysis, we evaluated a total of 108 patients with recurrent PC that received RGS of ^68^Ga-PSMA-11-PET/CT positive PC recurrences between April 2013 and September 2017. Patients’ characteristics were obtained by reviewing the medical records. After exclusion of patients with (i) only low dose CT imaging (defined with an x-ray tube current smaller than 80 mAs [24]), (ii) no LNM present in ^68^Ga-PSMA-11-PET/CT analysis (locoregional recurrence only), or (iii) a mismatch between ^68^Ga-PSMA-11-PET/CT positivity and histology (PET-positive LNs without positive histological correlate), 80 patients were used for further analyses (see Supplemental Figure 1 [25, 26]). The patients presented with biochemical recurrence following initial treatment (median PSA level before ^68^Ga-PSMA-11-PET/CT was 1.2 ng/ml, range 0.2–8.5 ng/ml) with a median Gleason score of 7b (range 6–9) (see Supplemental Table 1). Patients predominantly (96.3%) received RPE as initial treatment.

This evaluation was done upon written informed consent from all patients with the purpose of anonymized evaluation and publication. This investigation was approved by the Ethics Committee of the Technical University of Munich (TUM) (ERB 466/16 s).

### ^68^Ga-PSMA-11-PET/CT

All patients received diagnostic contrast-enhanced CT imaging during the late portal phase on a hybrid PET/CT scanner. Forty-seven patients received inhouse imaging using a Biograph mCT scanner after tracer injection of ^68^Ga-PSMA-11 ligand complex (mean 401 MBq; range 90–775 MBq). Thirty-three patients received ^68^Ga-PSMA-11-PET/CT scans at external institutions (see Supplemental Table 2 for CT types and acquisition parameters). Every imaging report was performed by an experienced nuclear medicine physician and a radiologist.

### Radioguided surgery

A detailed description of the applied technique was recently described elsewhere [13]. In short, suspicious LNs detected on ^68^Ga-PSMA-11-PET/CT imaging and adjacent non-diseased templates were selectively surgically resected guided by a gamma probe following intravenous application of radioactively labeled PSMA with ^99m^Technetium-PSMA-I&T (66 patients) or ^111^Indium-PSMA-I&S (14 patients). Ex vivo gamma measurements were performed to confirm successful removal of suspected lesions. At the end of surgery, remaining metastatic lesions were excluded by an additional round of gamma probe measurements in situ. Histological analysis was performed of all resected lymph node templates including PSMA expression analysis (monoclonal murine PSMA antibody [clone 3E6]; Dako, Hamburg, Germany). Correlation of imaging finding and histology was performed by anatomic location, ex vivo gamma probe measurements, LN size, and PSMA expression. In 87% of patients, there were only singular LNM or multiple LNMs had separate anatomic locations. In 13% of patients with two LNM in one resection template, lymph node diameter, and histopathology were assessed by a physician to correlate histology to imaging findings.

### LN segmentation

**Fig. 1:**
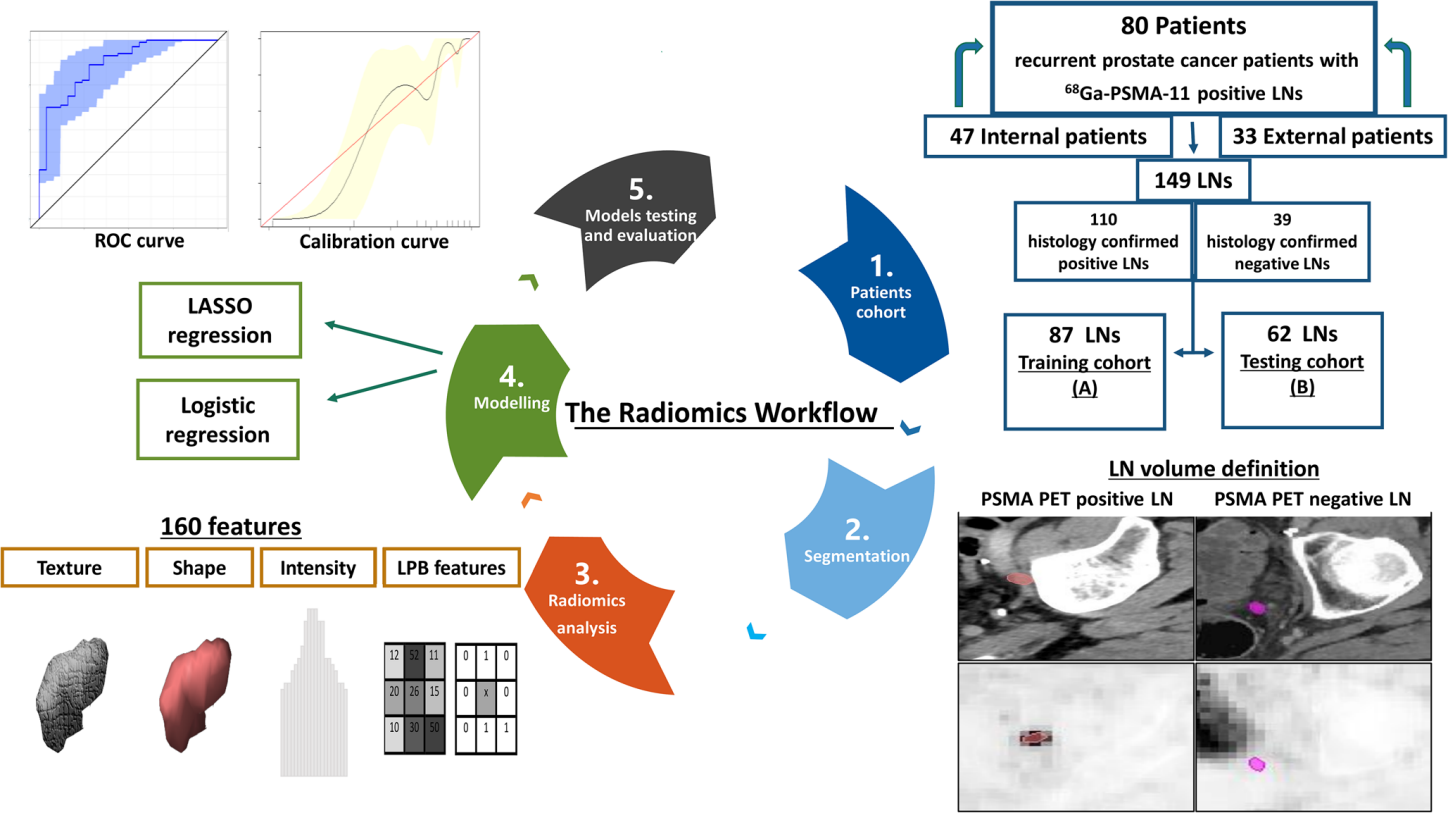
Schematic overview of the radiomics workflow. (1) Separation of a training patient cohort with internal imaging studies and a testing patient cohort with external imaging studies. (2) Manual segmentation of lymph nodes with a clear correlation to histology. (3) Preprocessing and radiomics feature extraction. (4) Modeling of prediction models using the least absolute shrinkage and selection operator (LASSO) for radiomic models and logistic regression for conventional CT parameters. (5) Testing and evaluation of model performance using receiver operator characteristic (ROC) curve, calibration curves and decision curve analysis

LN segmentation was conducted manually using Eclipse 13.0 (Varian Medical Systems, Palo Alto, USA) on the contrast-enhanced diagnostic CT datasets (see Fig. 1 for a depiction of the workflow). The segmentation was done by a radiation oncologist with 4 years of experience. First, LNs suspicious for LNM following the PSMA PET/CT report were segmented. In total, 832 LNs were found in the histological workup after RGS. Non-suspicious LNs were segmented on CT only if the LN (i) was visible on CT, and (ii) a direct correlation to the histological workup was possible. In total, 149 LNs with confirmed histology (110 histologically positive LNs, 39 histologically negative LNs) were analyzed. For the standardized uptake value (SUV) calculation, regions of interest were semi-automatically segmented using a 3D Slicer PET Tumor Segmentation module which applies the “just-enough-interaction” approach by Beichel et al. [27]. The focal maximum uptake was calculated using the 3D Slicer PET-IndiC extension.

The short diameter of all LNs was measured. All LNs were evaluated with an expert rating regarding likelihood of LNM by a blinded nuclear medicine physician with 5 years of experience.

Two separte blinded delineations per LN were performed by JP and MS in 20 patients from the training set (see Fig. 1) to compensate for operator-dependent segmentation bias (37 LNs). The patients were selected by random sampling stratified for LNM status. The Dice similarity coefficients (DSC) was calculated using the SlicerRT extension of 3D Slicer (3D Slicer, Version 4.8 stable release) [28].

### Radiomics features extraction

Radiomics feature and preprocessing was performed using the pyradiomics package (version 2.1) in Python (version 3.6.4) [25]. For preprocessing, a fixed bin width of 5 was used for image discretization to achieve a bin count between 16 and 128 [25, 29]. This resulted in a mean bin count of 37.

Isotropic resampling was performed to a voxel size of 1 × 1 × 1 mm using Bspline interpolation. Shape, first-order, and texture features were computed from the original image according the “image biomarker standardization initiative” (IBSI) guidelines [30]. Moreover, intensity features from local binary pattern (LBP) filtered images were calculated. Among other filtering methods, the LBP filter has not yet been defined by the IBSI. LBP-derived images were computed three-dimensionally using a level of one and two, as well as the kurtosis image. In total, 156 features were extracted. All extracted features are listed in Supplemental Table 3.

### ComBat batch harmonization

ComBatHarmonization has been proposed as a method for the correction of batch effects among radiomics multicenter cohorts [31, 32]. Its value to improve reproducibility between different centers has been shown in multiple studies [33–35]. Based on the given feature distribution it estimates the additive and multiplicative batch effects using a maximum likelihood approach. We applied parametric ComBat harmonization (https://github.com/Jfortin1/ComBatHarmonization, accessed April 16, 2020) correcting for PET/CT scanner models.

### Statistical analysis and model building

Statistical analysis and radiomic model building were performed using R (version 3.4.0, R core team, Vienna, Austria). All 47 patients that received inhouse PET/CT scans were used for training and validation (87 LNs). Prior to modeling, features susceptible to variances in segmentation in the 20 patients that received multiple independent segmentations (intraclass correlation coefficient (3,1) of < 0.8) were excluded.

For modeling, the least absolute shrinkage and selection operator (LASSO) was used. Using the “glmnet” package, the hyperparameter “lambda” was optimized for the prediction of histologically defined LNM status using 10-fold cross-validation in the training set. All 33 remaining patients that received external imaging were used as external test cohort (62 LNs). All over, we compared four distinct radiomic models: “Radiomics-texture,” “Radiomics-shape/intensity,” “Radiomics-LBP,” and a “Radiomics-combined” model. Texture, shape, intensity, and LBP features were used as input features for “Radiomics-combined.” For comparison, clinical parameters such as LN short diameter, LN volume, and expert evaluation (expert rating) were used as competing models. Prediction performance stability was evaluated by bootstrapping using the “fbroc” package (1000-fold). The final models were tested on the external test set. The optimal cutpoint for the radiomics model was determined using maximally selected rank statistics as implemented in the “maxstat” package on the training set. As a performance metric, the area under the receiver operator characteristic (ROC) curve (AUC) was calculated. Two models were compared using the rcorrp.cens function in the “Hmisc” package. Feature values and values were compared between two groups using the Wilcoxon rank-sum test. Correlation to LN volume was assessed using the Spearman’s rank correlation. A *p* value of < 0.05 was regarded as statistically significant. In cases of multiple testing, *p* values were adjusted using Bonferroni correction. Calibration curves were computed using the “gbm” package.

To compare the clinical net benefit, decision curve analysis was performed as described by Vickers et al. [https://www.mskcc.org/departments/epidemiology-biostatistics/biostatistics/decision-curve-analysis] [36]. The net benefit is calculated by subtracting the proportion of false-positive patients from the proportion of true-positive patients, weighted by the relative harm of a false-negative and false-positive result. The decision curves for “treating no patients” and “treating all patients” were depicted as reference.

## Results

Histologically positive LNs represented 70.1% and 79.0% in the training and test sets, respectively (see Table 1 for LN characteristics). The mean LN short diameter was generally short with 0.63 and 0.68 cm in the training and test set, respectively (see Supplemental Figure 2 for LN volume distribution).

**Table 1:** Patient and lymph node characteristics. Lymph node metastases status was determined by histological confirmation following radioguided surgery using ^99m^Technetium- or ^111^Indium-PSMA-Prostate specific membrane antigen (PSMA) radionuclides

	Training cohort (A)		Testing cohort (B)	
	Number	%	Number	%
Number of Lymph nodes	87	59.4%	62	41.6%
Number of patients	47	58.7%	33	41.3%
Age median (range)	69 years (49–78)		69 years (42–76)	
Histology				
Histology-confirmed positive LNs	61	70.1%	49	79%
Histology-confirmed negative LNs	26	29.9%	13	21%
Short-axis diameter (mm)				
Mean	63 mm		68 mm	
SD	± 28 mm		± 34 mm	
Range	(25–193 mm)		(32–170 mm)	
Volume (cm^3^)				
Mean	0.16 cm^3^		0.14 cm^3^	
SD	± 0.13 cm^3^		± 0.07 cm^3^	
Range	(0.06–1.04 cm^3^)		(0.05–0.51 cm^3^)	

The two independent segmentations overlapped with a median DSC of 0.89. Due to susceptibility to segmentation variances, 57%, 18%, 19%, and 15% of shape, intensity, texture, and LBP features were excluded, respectively.

In accordance with previous studies, 43% of all texture features were correlated to LN volume with a Spearman’s rank correlation coefficient of at least ± 0.6 [37–39]. Even more markedly, 78% of all shape features were correlated to volume. Only 6% of all intensity and 24% of all LBP features showed a correlation coefficient in the same range (see Supplemental Figures 3-6 for correlation coefficients of all features).

Next, the values of all 156 features were compared using the Wilcoxon rank-sum test in the total patient set. Eighty-four features showed a significantly different distribution between histologically positive and negative LNs (see Supplemental Table 4).

### LBP features outperform intensity, shape, and texture features

Among the radiomic models, Radiomics-texture, Radiomics-shape, and Radiomics-intensity showed similar predictive performances with AUCs of 0.78, 0.77, and 0.76 in the training cohort, and 0.78, 0.83, and 0.73 in the testing cohort, respectively. Radiomics-LBP achieved better predictive performance with AUC values of 0.86 in the training set and 0.90 in the testing set.

**Fig. 2:**
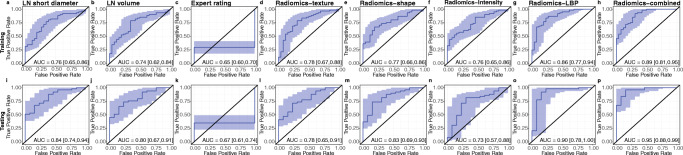
Receiver operator characteristic (ROC) curves for the prediction of lymph node metastases status. Depiction of ROC curves for all models in the training (top row) and test (bottom row) patient cohorts. The area under the ROC curve (AUC) is depicted. The blue areas represent the 95% confidence intervals

The model Radiomics-combined trained on all radiomic feature types further increased the discriminative performance to an AUC of 0.89 in the training set and an even better performance on the test set (AUC testing 0.95, +absolute difference 0.06). This was significantly better than Radiomics-texture, Radiomics-shape, and Radiomics-intensity (training: *p* = 0.01, *p* = 0.004, *p* = 0.006, respectively; testing: p = 0.004, *p* = 0.02, *p* = 0.01, respectively), but not Radiomics-LBP (training: *p* = 0.31, testing: *p* = 0.38). SUVmax as a guidance model for LN evaluation achieved AUC values of 0.98 (0.95–1.00) and 1.00 (1.00–1.00) in the training and test sets, respectively. See Table 2 for 95% confidence intervals (95% CIs), Fig. 2 for ROC curves, and Supplemental Figure 7 for calibration curves.

**Table 2:** Area under the receiver operator characteristic curve (AUC) values of the prediction models for lymph node metastases. Four radiomic models using different input features were compared with conventional measures. The respective AUC values and 95% confidence intervals (95% CI) are depicted

Model	Training cohort	Testing cohort
	*n* = 87 LN	*n* = 66 LN
	AUC (95% CI)	AUC (95% CI)
LN short diameter	0.76 (0.65–0.86)	0.84 (0.74–0.94)
LN Volume	0.74 (0.62–0.84)	0.80 (0.67–0.91)
Expert rating	0.65 (0.59–0.70)	0.67 (0.61–0.74)
Radiomics-texture	0.78 (0.67–0.88)	0.78 (0.65–0.91)
Radiomics-shape	0.77 (0.66–0.86)	0.83 (0.69–0.93)
Radiomics-intensity	0.76 (0.65–0.86)	0.73 (0.57–0.88)
Radiomics-LBP	0.86 (0.77–0.94)	0.90 (0.78–1.00)
Radiomics-combined	0.89 (0.81–0.95)	0.95 (0.88–0.99)

### Radiomics-combined showed superior predictive performance compared to conventional CT measures

Of all conventional parameters, *LN* short diameter achieved the best predictive performance (AUC training 0.76, AUC testing 0.84), followed by LN volume (AUC training 0.74, AUC testing 0.80) and expert rating (AUC training 0.65, AUC testing 0.67). In direct comparison, Radiomics-combined significantly outperformed LN short diameter (*p* = 0.002), LN volume (*p* = 0.001), and expert rating (*p* < 0.0001) on the training set. This was reproducible in the test set for all three conventional measures (expert rating (*p* < 0.0001), LN volume (*p* = 0.007), LN short diameter (*p* = 0.03)).

The conventional parameters and the best radiomic model (Radiomics-combined) were tested in logistic regression for LNM status on the test set (see Table 3). After adjustment for multiple testing, LN short diameter *(*odds ratio (OR) 5.6, *p* = 0.026)*,* and Radiomics-combined *(*OR 22, *p* = 0.0024) were the only significantly associated factors. In multivariate analysis of all three significant factors, only Radiomics-combined retained its significance (*p* = 0.0035). LN volume, LN short diameter, and the Radiomics-combined score also showed a significantly different distribution between histologically positive and negative LNs in the test set (*p* = 0.0005, *p* = 0.048, *p* < 0.0001, respectively, see Supplemental Figure 8).

**Table 3:** Univariate and multivariate analysis for histology confirmed lymph node recurrence. Univariate and multivariate logistic regression of the linear predictors of the conventional parameters and the best performing radiomics model (*Radiomics-combined*)

Clinical Variables	Univariate analysis			Multivariate analysis		
	Odds ratio	95% CI	*p* value	Odds ratio	95% CI	*p* value
Expert rating	2.7	0-Inf	1	–	–	–
LN short diameter	5.6	2–25	0.026*	2.34	0.59–15.2	0.34
LN Volume	9.8	2.1–8.6	0.08	–	–	–
Radiomics-combined	22	5.2–190	0.0024*	15.5	3.35–149	0.0035*

LN lymph node, Inf infinity. *Significant result

### Clinical usefulness of the Radiomics-combined model

On the test set, Radiomics-combined predicted LNM status with a balanced accuracy, and Matthews correlation coefficient (MCC) of 0.73 and 0.46, respectively (see Table 4 for more prediction metrics of all tested models). The best classification performance was achieved by Radiomics-LBP with a balanced Accuracy and MCC of 0.84 and 0.74, respectively. While limited in specificity, both models achieved good sensitivity, negative predictive value, and positive predictive value measures. LN short diameter split at a diameter of 0.8 cm predicted LNM status with a balanced accuracy and MCC of 0.67 and 0.29, respectively [5]. LN volume posed the best conventional model with a balanced accuracy of 0.73, but an inferior NPV.

**Table 4:** Classification metrics of all tested models. Four radiomic models using different input features were compared with conventional measures. The respective performance metrics (Matthews correlation coefficient (MCC), balanced accuracy, sensitivity, specificity, positive predictive value (PPV), and negative predictive value (NPV)) determined on the test set are reported

Model	MCC	Balanced accuracy	Sensitivity	Specificity	PPV	NPV
LN short diameter	0.32	0.67	0.35	1.00	1.00	0.29
LN Volume	0.37	0.72	0.67	0.77	0.92	0.38
Expert rating	0.32	0.67	0.35	1.00	1.00	0.29
Radiomics-texture	0.30	0.68	0.73	0.62	0.88	0.38
Radiomics-shape	0.37	0.73	0.61	0.85	0.94	0.37
Radiomics-intensity	0.25	0.57	0.98	0.15	0.81	0.67
Radiomics-LBP	0.74	0.84	0.98	0.69	0.92	0.9
Radiomics-combined	0.64	0.73	1.00	0.46	0.88	1.00

**Fig. 3:**
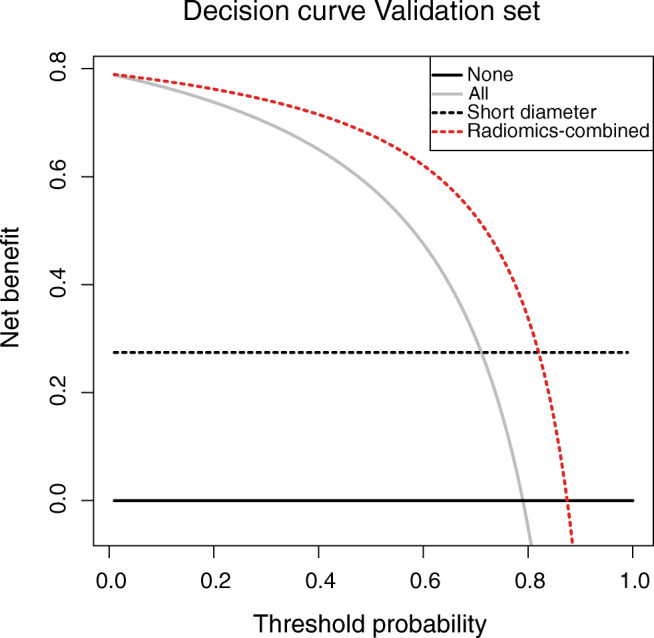
Lymph node–specific decision curve. The decision curve analysis plots the expected net benefit against the threshold capacity on the test set. The net benefit is calculated by subtracting the proportion of false-positive lymph nodes from the proportion of true-positive lymph nodes, weighted by the relative harm of a false-negative and false-positive result. The decision curves for “treating no lymph nodes” and “treating all lymph nodes” are depicted as reference. A decision model shows a clinical benefit if it achieves larger net benefit values than both reference strategies or any other model. This decision curve reflects the treatment decision on the lymph node level. The best radiomic model “Radiomics-combined” was split at an optimal cutpoint which was determined on the training set. It is compared to LN short diameter split at 0.8 cm

Decision curve analysis computed to reflect the treatment decision regarding a specific LN revealed a clinical net benefit of Radiomics-combined above the two alternative options “treat all lymph nodes” and “treat no lymph nodes” (see Fig. 3). Moreover, there was a larger net benefit than for LN short diameter between a threshold range of 0.0 and 0.9.

### Feature importance

Radiomics-combined was dominated by two LBP features “10th-percentile” and “inter-quartile-range” both computed from the kurtosis LBP image, and the shape feature “SurfaceVolumeRatio” (see Supplemental Table 5 for a feature importance ranking of all radiomic models). These features were also the most important in their respective single feature class models (Radiomics-LBP and Radiomics-shape), respectively.

### Influence of ComBat harmonization

The results reported above were based on radiomic features after ComBat harmonization. The omission of ComBat harmonization led only to small changes in model performance. The performance of the best model Radiomics-combined decreased by an AUC value of 0.01 without marked change in contingency table measures or logistic regression analyses (see Supplemental Tables 6, 7, and 8).

## Discussion

In this work, we have developed CT-based radiomic models for the detection of LNMs. A patient cohort that received RGS for recurrent PC was used providing histological correlates as gold standard for all analyzed LNs. The combined radiomic model achieved the best predictive performance of all radiomic models and significantly outperformed conventional CT measures. The model retained significant correlation to LNM status in multivariate analyses and showed a larger clinical net benefit than LN short diameter.

A few previous studies have analyzed the value of computational feature extraction of LNs to predict LNM status. In non-small-cell lung cancer patients, Flechsig et al. could demonstrate that LN median intensity (“density”) was significantly different between histologically confirmed LNMs and LNs on non-contrast-enhanced CT. On contrast-enhanced CT there was only a trend towards significance [40, 41]. Further on, with an AUC value of 0.82, it showed better predictive performance than short-axis diameter (AUC 0.65). Giesel et al. performed a similar analysis including 40 PC patients. The authors could demonstrate a correlation of LN density to PSMA PET/CT positivity although without histological confirmation [42]. Our approach differed in several ways from the studies discussed above. First, we used diagnostic contrast-enhanced CT imaging data which is regularly used in the clinic. Secondly, following the radiomics concept, we extracted a large number of radiomic features enabling us to filter the most relevant features.

One further study has performed radiomic analysis for LNM detection in cervical cancer patients on the basis of T2-weigthed MRI imaging. The resulting radiomic model achieved similar a predictive performance for LNM status in an internal validation cohort with an AUC of 0.85 [43]. Our best model achieved a predictive performance with an AUC of 0.95 in the test set. This performance was significantly better than all conventional CT measures including the current clinical standard “LN short diameter”.

Previous studies have highlighted the large dependency of texture features to the VOI volume especially for small volume sizes [37–39]. Our study could demonstrate that a large number of texture and shape features indeed correlated with volume. Moreover, both feature types achieved a predictive performance for the prediction of LNM status which was comparable to LN volume alone. This may indicate that texture and shape features have only a limited incremental value in small VOIs. In contrast, LBP features achieved good predictive performances demonstrating their value despite small VOI volumes.

LBP features have not yet been frequently used in radiomic studies. LBP constitutes an image filter in which each voxel is labeled according to its relationship between the gray levels towards surrounding voxels. Each surrounding voxel receives a binary label representing a higher or lower intensity value. Each unique label combination of the surrounding voxels is allocated a new gray value [44]. Different LBP values may, thus, represent semantic properties such as edges. The two most important features in our combined radiomics mode were LBP features underlining the value of LBP features for the analysis of small volume VOIs.

Our radiomic models outperformed all conventional CT-based models. However, SUVmax showed the highest predictive value of all models (AUC training 0.98, AUC-testing 1.00). These results are biased by the fact that the PSMA tracer uptake was used to define LNs suspicious to be LNM before RGS. Moreover, PET-positive LNs without positive histological correlate were excluded to increase the reliability of the histological finding for the training of CT models. As a consequence, this study design does not allow a comparison of the CT-based models with the performance of PSMA-uptake. Due to this selection bias, all reported performances cannot be set equal to the performance in unselected cases. Still, currently, PSMA PET/CT remains the optimal choice for pre-therapeutic assessment of lymph node involvement.

We performed a decision curve analysis demonstrating a clinical net benefit of the Radiomics-combined model. This analysis was done on a LN-specific basis. Thus, it evaluates the treatment decision to include a LN into a targeted treatment or not. A CT-based LMM detection model could be applied to the optimization of SRT planning in the absence of PSMA PET/CT imaging. First, the CTV could be extended to cover atypically located LNs that are classified as LNM on planning CTs. Secondly, suspected LNM could be treated with the simultaneous integrated boost technique resulting in larger radiation doses. On the contrary, the model predictions may also alter the overall choice of treatment (e.g. salvage PLND). This model was generated in recurrent PC patients. However, it may also be valuable in the primary treatment setting for decision support or guidance of the treatment planning.

Our study bears several relevant limitations. First, histological confirmation of PSMA-ligand positive LNs was facilitated by RGS. However, despite the use of RGS, systematic template resection, and thorough comparison between imaging and LNs described in the pathology report single misclassifications between imaging and pathology could still be possible. To minimize this bias, we decided to exclude all LNs positive in ^68^Ga-PSMA-11 imaging without a positive histological correlate. Secondly, only a small number of negative LNs could be segmented as we focused on LNs that had a clear histological correlate and that were situated in the pelvis. In fact, a large number of only a few millimeters measuring LNs found in histology could not be found on CT imaging. Thirdly, the developed models were validated using only a “quasi-external” test set. All of these patients received imaging at multiple external facilities with diverse scanner types and acquisition protocols. On the other hand, all patients received RGS and histological workup at one institution. Interestingly, the radiomic model showed good reproducibility between both cohorts. To achieve optimal proof for generalizability, a completely independent external validation should be performed. Moreover, the analyzed cohort was largely biased by the fact that PET imaging was used for LN selection, that only LN visible on CT could be segmented, and that LNs with a mismatch between histology and imaging were excluded. As a consequence, the relatively high AUC values may be overestimated and not representative. To reach a sample number sufficient for radiomics analysis, multiple LNs were included per patient. It should be noted that correlation in the radiomics phenotype of LNs from the same patients may have impeded optimal model development. Finally, the study was conducted in a retrospective fashion. A future prospective RGS trial could be used to validate the developed radiomics model with histological confirmation. Despite these limitations, our study achieved a radiomics quality score of 53% (see Supplemental Table 7) [45]. This score was higher than in 97% of studies analyzed in a recent review [46].

To conclude, we were able to develop a CT-based radiomic model for the detection of LNMs. All included LNs were histologically confirmed by PSMA radioguided surgery. The combined radiomic model, based on texture, shape, intensity and local binary pattern features significantly outperformed conventional measures in predictive performance and showed a clinical net benefit above the short diameter of LNs. All models were validated using external imaging studies. A future validated model could be used to provide guidance for personalization of therapy in case of unavailability of PSMA PET/CT imaging or in cases of PSMA PET/CT indeterminate LNs. In case of availability, PSMA PET/CT remains unmatched in diagnostic capacity.

## Electronic supplementary material

ESM 1 (DOCX 1.68 MB).

## Data Availability

The datasets analyzed during this study are available from the corresponding author on reasonable request depending on ERB approval.

## Abbreviations

95% CI: 95% confidence interval
AUC: Area under the curve
CT: Computed tomography
CTV: Clinical target volume
LASSO: Least absolute shrinkage and selection operator
LBP: Local binary patterns
LNM: Lymph nodes metastasis
LN: Lymph node
NPV: Negative predictive value
PC: Prostate carcinoma
PET: Positron emission tomography
PLND: Pelvic lymph node dissection
PSMA: Prostate-specific membrane antigen
RGS: Radioguided surgery
ROC: Receiver operator characteristic
RPE: Radical prostatectomy
RTOG: Radiation Therapy Oncology Group
SRT: Salvage radiotherapy
SUV: Standardized uptake value
VOI: Volume of interest
