# Supplementary material for: A CT-based radiomics model to detect prostate cancer lymph node metastases in PSMA radioguided surgery patients
Source: Eur J Nucl Med Mol Imaging. 2020 May 28;47(13):2968–77. doi: 10.1007/s00259-020-04864-1 (PMC7680305; doi:10.1007/s00259-020-04864-1)
Supplement: Supplementary file 1 — (DOCX 1.68 MB). [file 259_2020_4864_MOESM1_ESM.docx]

**Supplemental Material**

[Supplemental Tables 2](#_Toc40445883)

[SUPPLEMENTAL TABLE 1 2](#_Toc40445884)

[SUPPLEMENTAL TABLE 2 3](#_Toc40445885)

[SUPPLEMENTAL TABLE 3 4](#_Toc40445886)

[SUPPLEMENTAL TABLE 4 8](#_Toc40445887)

[SUPPLEMENTAL TABLE 5 11](#_Toc40445888)

[SUPPLEMENTAL TABLE 6 12](#_Toc40445889)

[SUPPLEMENTAL TABLE 7 13](#_Toc40445890)

[SUPPLEMENTAL TABLE 8 14](#_Toc40445891)

[SUPPLEMENTAL TABLE 9 15](#_Toc40445892)

[SUPPLEMENTAL FIGURES 17](#_Toc40445893)

[SUPPLEMENTAL FIGURE 1 17](#_Toc40445894)

[SUPPLEMENTAL FIGURE 2 18](#_Toc40445895)

[SUPPLEMENTAL FIGURE 3 19](#_Toc40445896)

[SUPPLEMENTAL FIGURE 4 20](#_Toc40445897)

[SUPPLEMENTAL FIGURE 5 21](#_Toc40445898)

[SUPPLEMENTAL FIGURE 6 22](#_Toc40445899)

[SUPPLEMENTAL FIGURE 7 23](#_Toc40445900)

[SUPPLEMENTAL FIGURE 8 24](#_Toc40445901)

[References 25](#_Toc40445902)

# Supplemental Tables

## SUPPLEMENTAL TABLE 1

**Patient demographics**

| **Patients** | **All** | **Training**  **Cohort** | **Testing**  **Cohort** |
| --- | --- | --- | --- |
| **Characteristics** | **N = 80 (100%)** | **N = 47 (100%)** | **N = 33 (100%)** |
| **Age (years)** |  |  |  |
| Median | 69 | 69 | 69 |
| Range | (42-78) | (49–78) | (42–76) |
| **Tumor stage** |  |  |  |
| <pT2c | 2           (2.5%) | 1         (2.1%) | 1         (3%) |
| pT2c | 24         (30%) | 11       (23.4%) | 13       (39.4%) |
| pT3a | 26         (32.5%) | 15       (31.9%) | 11       (33.3%) |
| pT3b | 23         (28.8%) | 16       (34%) | 7         (21.2%) |
| pT4 | 0           (0%) | 0         (0%) | 0         (0%) |
| Not available | 5           (6.2%) | 4         (4.3%) | 1         (3%) |
| **Initial PSA (ng/ml)** |  |  |  |
| Mean | 15,2 | 15,5 | 15 |
| Median | 10.2 | 10.2 | 12.3 |
| Range | (0.65-65) | (0.65-48.8) | (5-45.3) |
| **Initial Gleason score** |  |  |  |
| 5 | 4           (5%) | 3          (6.4%) | 1         (3%) |
| 6 | 8           (10%) | 5          (10.6%) | 3         (9.1%) |
| 7a | 13         (16.3%) | 8         (17%) | 5         (15.1%) |
| 7b | 20         (25%) | 9         (19.1%) | 11       (33.3%) |
| 8 | 8           (10%) | 6         (12.8%) | 2         (6.1%) |
| 9 | 19         (20%) | 11       (23.4%) | 8         (24.2%) |
| 10  Not available | 0           (0%)  8           (10%) | 0         (0%)  5         (10.6%) | 0         (0%)  3         (9.1%) |
| **International Society of Urological Pathology (ISUP) scoring** |  |  |  |
| 1 | 12         (15%) | 8         (17%) | 4         (12.1%) |
| 2 | 13         (16.3%) | 8         (17%) | 5         (15.1%) |
| 3 | 20         (25%) | 9         (19.1%) | 11       (33.3%) |
| 4 | 8           (10%) | 6         (12.8%) | 2         (6.1%) |
| 5 | 19         (20%) | 11       (23.4%) | 8         (24.2%) |
| Not available | 8           (10%) | 5         (10.6%) | 3         (9.1%) |
| **Initial Treatment** |  |  |  |
| Radical prostatectomy (RP) alone  Radiation therapy (RT) alone | 40         (50%)  3           (3.8%) | 26       (55.3%)  3         (6.4%) | 14       (42.4%)  0         (0%) |
| RP plus adjuvant RT | 37         (46.3%) | 18       (38.3%) | 19       (57.8%) |
| **Previously received treatment before ^68^Ga-PSMA-11-PET/CT** |  |  |  |
| Radical prostatectomy (RP)  Radiation therapy (RT) | 77         (96.3%)  42         (52.5%) | 44       (93.6%)  23       (48.9%) | 33       (100%)  19       (57.6%) |
| Systemic therapy (Hormon therpy or/and Chemotherapy) | 10         (12.5%) | 5         (10.6%) | 5         (15.2%) |
| **Radioactively labelled PSMA used during Radio guided surgery** |  |  |  |
| ^99m^Technetium-PSMA-I&S | 66         (82.5%) | 36       (76.6%) | 30       (90.9%) |
| ^111^Indium-PSMA-I&T | 14         (17.5%) | 11       (23.4%) | 3         (9.1%) |
| **PSA prior to ^68^Ga-PSMA-11- PET/CT  (ng/ml)** |  |  |  |
| Mean | 1.31 | 1.30 | 1.45 |
| Median | 1.15 | 1.08 | 1.79 |
| Range | (0.23-8.5) | (0.23-8.5) | (0.27-6.04) |

## SUPPLEMENTAL TABLE 2

**CT acquisition Parameters**

|  | Training | Testset |
| --- | --- | --- |
| Scanner type | Siemens Biograph: 27  Siemens Somatom: Definition 20 | Siemens Biograph: 27  Siemens Somatom Definition:  4  Philips Gemini TF TOF: 3 |
| Matrix | 512 x 512 pixel | 512 x 512 pixel |
| Pixel Spacing | Median: 0,75 x 0,75  Range (0,59 x 0,59) to (1,52 X 1,52) | Median: 0,87 x 0,87  Range (0,65 x 0,65) to (2,64 X 2,64) |
| Slice thickness | Median 5  Range (5-5) | Median 4  Range (1,5-7) |
| Kernel | B30f: 1  I30f: 46 | B: 3  B19f: 1  B25f: 1  B30f: 8  B31f: 9  B70f: 2  I30f: 7  I31f: 1  Not available: 1 |
| Voltage | 120 KVP | 120 KVP |
| X ray tube Current | 398,5  (94-650) | Median 362,0  (97-642) |
| Image Reconstruction | Primary axial ct_som 5 spi: 47 | Derived secondary axial average: 1  Primary axial ct_som 5 spi: 32 |

Abbreviations: CT: computed tomography, PET: positron emission tomography

Manufacturer: Philips (Amsterdam, Netherlands), Siemens (Munich, Germany):

## SUPPLEMENTAL TABLE 3

**Extracted radiomics features (n=156)**

All extracted features were computed according the “image biomarker standardization initiative” (IBSI) guidelines (1). The pyradiomics package (version 2.0) implemented in python (version 3.6.4) was used for feature extraction (2). For preprocessing, a fixed bin width of 5 was used for image discretization. Isotropic resampling was performed to a voxel size of 1x1x1 mm using Bspline interpolation.

|  | **Shape Features** |
| --- | --- |
| 1.) | Volume |
| 2.) | Surface Area |
| 3.) | Surface Volume Area |
| 4.) | Sphericity |
| 5.) | Spherical Disproportion |
| 6.) | Maximum 3D Diameter |
| 7.) | Maximum 2D Diameter Slice |
| 8.) | Maximum 2D Diameter Column |
| 9.) | Maximum 2D Diameter Row |
| 10.) | Major Axis |
| 11.) | Minor Axis |
| 12.) | Least Axis |
| 13.) | Elongation |
| 14.) | Flatness |
|  | **First Order Features** |
| 1.) | Energy |
| 2.) | Intensity Histogram Entropy |
| 3.) | Minimum |
| 4.) | 10th Percentile |
| 5.) | 90th Percentile |
| 6.) | Maximum |
| 7.) | Mean |
| 8.) | Median |
| 9.) | Interquartile Range |
| 10.) | Range |
| 11.) | Mean Absolute Deviation (MAD) |
| 12.) | Robust Mean Absolute Deviation (rMAD) |
| 13.) | Root Mean Squared (RMS) |
| 14.) | Skewness |
| 15.) | Excess Kurtosis |
| 16.) | Variance |
| 17.) | Intensity Histogram Uniformity |
|  | **Local Binary pattern (LBP) Features-m1** |
| 1.) | Energy |
| 2.) | Intensity Histogram Entropy |
| 3.) | Minimum |
| 4.) | 10th Percentile |
| 5.) | 90th Percentile |
| 6.) | Maximum |
| 7.) | Mean |
| 8.) | Median |
| 9.) | Interquartile Range |
| 10.) | Range |
| 11.) | Mean Absolute Deviation (MAD) |
| 12.) | Robust Mean Absolute Deviation (rMAD) |
| 13.) | Root Mean Squared (RMS) |
| 14.) | Skewness |
| 15.) | Excess Kurtosis |
| 16.) | Variance |
| 17.) | Intensity Histogram Uniformity |
|  | **Local Binary pattern (LBP) Features-m2** |
| 1.) | Energy |
| 2.) | Intensity Histogram Entropy |
| 3.) | Minimum |
| 4.) | 10th Percentile |
| 5.) | 90th Percentile |
| 6.) | Maximum |
| 7.) | Mean |
| 8.) | Median |
| 9.) | Interquartile Range |
| 10.) | Range |
| 11.) | Mean Absolute Deviation (MAD) |
| 12.) | Robust Mean Absolute Deviation (rMAD) |
| 13.) | Root Mean Squared (RMS) |
| 14.) | Skewness |
| 15.) | Excess Kurtosis |
| 16.) | Variance |
| 17.) | Intensity Histogram Uniformity |
|  | **Local Binary pattern (LBP) Features-kurtosis** |
| 1.) | Energy |
| 2.) | Intensity Histogram Entropy |
| 3.) | Minimum |
| 4.) | 10th Percentile |
| 5.) | 90th Percentile |
| 6.) | Maximum |
| 7.) | Mean |
| 8.) | Median |
| 9.) | Interquartile Range |
| 10.) | Range |
| 11.) | Mean Absolute Deviation (MAD) |
| 12.) | Robust Mean Absolute Deviation (rMAD) |
| 13.) | Root Mean Squared (RMS) |
| 14.) | Skewness |
| 15.) | Excess Kurtosis |
| 16.) | Variance |
| 17.) | Intensity Histogram Uniformity |
|  | **Gray Level Co-occurrence Matrix (GLCM) Features** |
| 1.) | Autocorrelation |
| 2.) | Joint Average |
| 3.) | Cluster Prominence |
| 4.) | Cluster Shade |
| 5.) | Cluster Tendency |
| 6.) | Contrast |
| 7.) | Correlation |
| 8.) | Difference Average |
| 9.) | Difference Entropy |
| 10.) | Difference Variance |
| 11.) | Joint Energy (IBSI: Angular Second Moment) |
| 12.) | Joint Entropy |
| 13.) | Informal Measure of Correlation (IMC) 1 |
| 14.) | Informal Measure of Correlation (IMC) 2 |
| 15.) | Inverse Difference Moment (IDM) |
| 16.) | Inverse Difference Moment Normalized (IDMN) |
| 17.) | Inverse Difference (ID) |
| 18.) | Inverse Difference Normalized (IDN) |
| 19.) | Inverse Variance |
| 20.) | Maximum Probability (IBSI: Joint maximum) |
| 21.) | Sum Entropy |
| 22.) | Sum of Squares (IBSI: Sum of Squares) |
| 23.) | Maximal Correlation Coefficient (MCC) |
|  | **Gray Level Size Zone Matrix (GLSZM) Features** |
| 1.) | Small Area Emphasis (SAE) |
| 2.) | Large Area Emphasis (LAE) |
| 3.) | Gray Level Non-Uniformity (GLN) |
| 4.) | Gray Level Non-Uniformity Normalized (GLNN) |
| 5.) | Size-Zone Non-Uniformity (SZN) |
| 6.) | Size-Zone Non-Uniformity Normalized (SZNN) |
| 7.) | Zone Percentage (ZP) |
| 8.) | Gray Level Variance (GLV) |
| 9.) | Zone Variance (ZV) |
| 10.) | Zone Entropy (ZE) |
| 11.) | Low Gray Level Zone Emphasis (LGLZE) |
| 12.) | High Gray Level Zone Emphasis (HGLZE) |
| 13.) | Small Area Low Gray Level Emphasis (SALGLE) |
| 14.) | Small Area High Gray Level Emphasis (SAHGLE) |
| 15.) | Large Area Low Gray Level Emphasis (LALGLE) |
| 16.) | Large Area High Gray Level Emphasis (LAHGLE) |
|  | **Gray Level Run Length Matrix (GLRLM) Features** |
| 1.) | Short Run Emphasis (SRE) |
| 2.) | Long Run Emphasis (LRE) |
| 3.) | Gray Level Non-Uniformity (GLN) |
| 4.) | Gray Level Non-Uniformity Normalized (GLNN) |
| 5.) | Run Length Non-Uniformity (RLN) |
| 6.) | Run Length Non-Uniformity Normalized (RLNN) |
| 7.) | Run Percentage (RP) |
| 8.) | Gray Level Variance (GLV) |
| 9.) | Run Variance (RV) |
| 10.) | Run Entropy (RE) |
| 11.) | Low Gray Level Run Emphasis (LGLRE) |
| 12.) | High Gray Level Run Emphasis (HGLRE) |
| 13.) | Short Run Low Gray Level Emphasis (SRLGLE) |
| 14.) | Short Run High Gray Level Emphasis (SRHGLE) |
| 15.) | Long Run Low Gray Level Emphasis (LRLGLE) |
| 16.) | Long Run High Gray Level Emphasis (LRHGLE) |
|  | **Neighbouring Gray Tone Difference Matrix (NGTDM) Features** |
| 1.) | Coarseness |
| 2.) | Contrast |
| 3.) | Busyness |
| 4.) | Complexity |
| 5.) | Strength |
|  | **Gray Level Dependence Matrix (GLDM) Features** |
| 1.) | Small Dependence Emphasis (SDE) |
| 2.) | Large Dependence Emphasis (LDE) |
| 3.) | Gray Level Non-Uniformity (GLN) |
| 4.) | Dependence Non-Uniformity (DN) |
| 5.) | Dependence Non-Uniformity Normalized (DNN) |
| 6.) | Gray Level Variance (GLV) |
| 7.) | Dependence Variance (DV) |
| 8.) | Dependence Entropy (DE) |
| 9.) | Low Gray Level Emphasis (LGLE) |
| 10.) | High Gray Level Emphasis (HGLE) |
| 11.) | Small Dependence Low Gray Level Emphasis (SDLGLE) |
| 12.) | Small Dependence High Gray Level Emphasis (SDHGLE) |
| 13.) | Large Dependence Low Gray Level Emphasis (LDLGLE) |
| 14.) | Large Dependence High Gray Level Emphasis (LDHGLE) |

## SUPPLEMENTAL TABLE 4

**Results of Wilcoxon Rank sum test for feature comparison on the total patient set.**

Radiomic feature values were compared between histologically defined positive and negative LNs using the Wilcoxon rank-sum test on the complete patient set (training and testing combined). Adjustment of p-values was performed using the Bonferroni method.

| Radiomic feature | p-value | Adjusted p-value |
| --- | --- | --- |
| lbp.3D.m2_firstorder_RobustMeanAbsoluteDeviation | 1,60E-09 | 2,50E-07 |
| lbp.3D.m2_firstorder_InterquartileRange | 2,60E-09 | 4,10E-07 |
| lbp.3D.m2_firstorder_MeanAbsoluteDeviation | 8,30E-09 | 1,30E-06 |
| lbp.3D.k_firstorder_10Percentile | 2,80E-08 | 4,40E-06 |
| lbp.3D.m2_firstorder_Uniformity | 3,10E-08 | 4,80E-06 |
| lbp.3D.m2_firstorder_ExcessKurtosis | 3,80E-08 | 5,90E-06 |
| original_shape_Maximum2DDiameterSlice | 4,10E-08 | 6,40E-06 |
| original_gldm_LargeDependenceLowGrayLevelEmphasis | 5,40E-08 | 8,40E-06 |
| lbp.3D.m2_firstorder_Entropy | 5,90E-08 | 9,20E-06 |
| lbp.3D.m2_firstorder_90Percentile | 6,50E-08 | 1,00E-05 |
| lbp.3D.m2_firstorder_Variance | 7,20E-08 | 1,10E-05 |
| lbp.3D.m2_firstorder_Median | 9,00E-08 | 1,40E-05 |
| original_glrlm_LongRunLowGrayLevelEmphasis | 1,10E-07 | 1,70E-05 |
| original_gldm_LowGrayLevelEmphasis | 1,10E-07 | 1,70E-05 |
| original_glrlm_LowGrayLevelRunEmphasis | 1,20E-07 | 1,90E-05 |
| original_glrlm_ShortRunLowGrayLevelEmphasis | 1,30E-07 | 2,00E-05 |
| original_shape_LeastAxisLength | 1,80E-07 | 2,80E-05 |
| original_firstorder_Energy | 1,80E-07 | 2,80E-05 |
| lbp.3D.m2_firstorder_RootMeanSquared | 1,80E-07 | 2,80E-05 |
| lbp.3D.m2_firstorder_Energy | 1,90E-07 | 3,00E-05 |
| original_shape_SurfaceVolumeRatio | 2,30E-07 | 3,60E-05 |
| original_glszm_ZoneEntropy | 5,90E-07 | 9,20E-05 |
| lbp.3D.m2_firstorder_Mean | 7,40E-07 | 1,20E-04 |
| original_glrlm_LongRunHighGrayLevelEmphasis | 8,40E-07 | 1,30E-04 |
| lbp.3D.m1_firstorder_Energy | 1,30E-06 | 2,00E-04 |
| original_shape_MinorAxisLength | 1,40E-06 | 2,20E-04 |
| original_gldm_HighGrayLevelEmphasis | 1,60E-06 | 2,50E-04 |
| original_shape_MeshVolume | 1,70E-06 | 2,70E-04 |
| original_glrlm_HighGrayLevelRunEmphasis | 1,70E-06 | 2,70E-04 |
| original_glszm_LargeAreaHighGrayLevelEmphasis | 1,70E-06 | 2,70E-04 |
| lbp.3D.m2_firstorder_Maximum | 1,80E-06 | 2,80E-04 |
| original_glszm_LowGrayLevelZoneEmphasis | 1,80E-06 | 2,80E-04 |
| original_gldm_DependenceNonUniformity | 1,80E-06 | 2,80E-04 |
| original_gldm_LargeDependenceHighGrayLevelEmphasis | 1,80E-06 | 2,80E-04 |
| original_glcm_JointAverage | 2,00E-06 | 3,10E-04 |
| original_shape_VoxelVolume | 2,10E-06 | 3,30E-04 |
| original_glcm_Autocorrelation | 2,10E-06 | 3,30E-04 |
| original_glrlm_ShortRunHighGrayLevelEmphasis | 2,10E-06 | 3,30E-04 |
| original_glszm_SizeZoneNonUniformity | 2,70E-06 | 4,20E-04 |
| original_glszm_HighGrayLevelZoneEmphasis | 2,90E-06 | 4,50E-04 |
| original_glrlm_RunLengthNonUniformity | 3,30E-06 | 5,10E-04 |
| lbp.3D.m1_firstorder_Maximum | 4,30E-06 | 6,70E-04 |
| original_gldm_DependenceEntropy | 5,50E-06 | 8,60E-04 |
| lbp.3D.m1_firstorder_Range | 5,90E-06 | 9,20E-04 |
| original_shape_SurfaceArea | 7,50E-06 | 1,20E-03 |
| original_firstorder_90Percentile | 7,70E-06 | 1,20E-03 |
| lbp.3D.k_firstorder_Maximum | 7,50E-06 | 1,20E-03 |
| original_gldm_SmallDependenceLowGrayLevelEmphasis | 7,90E-06 | 1,20E-03 |
| lbp.3D.m2_firstorder_Range | 8,70E-06 | 1,40E-03 |
| original_firstorder_Mean | 1,00E-05 | 1,60E-03 |
| original_firstorder_Maximum | 1,20E-05 | 1,90E-03 |
| original_firstorder_Median | 1,30E-05 | 2,00E-03 |
| lbp.3D.m1_firstorder_90Percentile | 1,40E-05 | 2,20E-03 |
| lbp.3D.k_firstorder_Energy | 1,50E-05 | 2,30E-03 |
| original_glcm_JointEntropy | 1,50E-05 | 2,30E-03 |
| original_glcm_Idmn | 1,60E-05 | 2,50E-03 |
| lbp.3D.m1_firstorder_RootMeanSquared | 1,80E-05 | 2,80E-03 |
| original_glszm_SmallAreaHighGrayLevelEmphasis | 2,30E-05 | 3,60E-03 |
| original_glrlm_RunEntropy | 2,60E-05 | 4,10E-03 |
| lbp.3D.k_firstorder_Range | 2,80E-05 | 4,40E-03 |
| original_glcm_Idn | 3,50E-05 | 5,50E-03 |
| original_glszm_GrayLevelNonUniformity | 3,80E-05 | 5,90E-03 |
| original_ngtdm_Coarseness | 3,80E-05 | 5,90E-03 |
| original_firstorder_RootMeanSquared | 5,30E-05 | 8,30E-03 |
| original_glszm_SmallAreaLowGrayLevelEmphasis | 5,40E-05 | 8,40E-03 |
| original_glcm_JointEnergy | 8,60E-05 | 1,30E-02 |
| lbp.3D.m1_firstorder_MeanAbsoluteDeviation | 9,20E-05 | 1,40E-02 |
| lbp.3D.m1_firstorder_Mean | 8,70E-05 | 1,40E-02 |
| lbp.3D.m1_firstorder_Variance | 9,90E-05 | 1,50E-02 |
| lbp.3D.m1_firstorder_Median | 1,10E-04 | 1,70E-02 |
| original_firstorder_Range | 1,20E-04 | 1,90E-02 |
| lbp.3D.m1_firstorder_InterquartileRange | 1,20E-04 | 1,90E-02 |
| lbp.3D.m1_firstorder_RobustMeanAbsoluteDeviation | 1,20E-04 | 1,90E-02 |
| lbp.3D.k_firstorder_Mean | 1,20E-04 | 1,90E-02 |
| original_glcm_SumEntropy | 1,20E-04 | 1,90E-02 |
| original_glcm_MaximumProbability | 1,30E-04 | 2,00E-02 |
| original_glrlm_GrayLevelNonUniformity | 1,40E-04 | 2,20E-02 |
| lbp.3D.k_firstorder_Median | 1,50E-04 | 2,30E-02 |
| original_gldm_GrayLevelNonUniformity | 1,60E-04 | 2,50E-02 |
| lbp.3D.m1_firstorder_Entropy | 1,70E-04 | 2,70E-02 |
| lbp.3D.k_firstorder_RootMeanSquared | 2,00E-04 | 3,10E-02 |
| original_glszm_GrayLevelNonUniformityNormalized | 2,20E-04 | 3,40E-02 |
| lbp.3D.m1_firstorder_Uniformity | 2,30E-04 | 3,60E-02 |
| original_glcm_Imc1 | 2,30E-04 | 3,60E-02 |
| original_glcm_ClusterProminence | 4,10E-04 | 6,40E-02 |
| original_gldm_SmallDependenceHighGrayLevelEmphasis | 4,40E-04 | 6,90E-02 |
| original_shape_Flatness | 5,20E-04 | 8,10E-02 |
| original_firstorder_Entropy | 6,60E-04 | 1,00E-01 |
| original_glcm_Correlation | 8,00E-04 | 1,20E-01 |
| lbp.3D.k_firstorder_ExcessKurtosis | 8,50E-04 | 1,30E-01 |
| original_ngtdm_Complexity | 9,80E-04 | 1,50E-01 |
| lbp.3D.k_firstorder_Entropy | 1,10E-03 | 1,70E-01 |
| lbp.3D.k_firstorder_Uniformity | 1,10E-03 | 1,70E-01 |
| original_firstorder_10Percentile | 1,40E-03 | 2,20E-01 |
| original_shape_Elongation | 2,00E-03 | 3,10E-01 |
| original_glrlm_GrayLevelNonUniformityNormalized | 2,00E-03 | 3,10E-01 |
| original_glszm_GrayLevelVariance | 2,20E-03 | 3,40E-01 |
| original_shape_Maximum3DDiameter | 2,40E-03 | 3,70E-01 |
| original_glszm_LargeAreaLowGrayLevelEmphasis | 2,50E-03 | 3,90E-01 |
| original_glcm_Imc2 | 2,80E-03 | 4,40E-01 |
| original_firstorder_Uniformity | 3,00E-03 | 4,70E-01 |
| original_glcm_ClusterTendency | 3,80E-03 | 5,90E-01 |
| lbp.3D.k_firstorder_Skewness | 4,80E-03 | 7,50E-01 |
| original_glrlm_GrayLevelVariance | 5,20E-03 | 8,10E-01 |
| original_gldm_GrayLevelVariance | 6,20E-03 | 9,70E-01 |
| original_firstorder_ExcessKurtosis | 6,30E-03 | 9,80E-01 |
| original_shape_MajorAxisLength | 2,10E-02 | 1 |
| original_shape_Maximum2DDiameterColumn | 2,30E-02 | 1 |
| original_shape_Maximum2DDiameterRow | 8,00E-03 | 1 |
| original_shape_Sphericity | 4,40E-01 | 1 |
| original_firstorder_InterquartileRange | 5,70E-02 | 1 |
| original_firstorder_MeanAbsoluteDeviation | 1,70E-02 | 1 |
| original_firstorder_Minimum | 8,40E-01 | 1 |
| original_firstorder_RobustMeanAbsoluteDeviation | 5,90E-02 | 1 |
| original_firstorder_Skewness | 1,80E-02 | 1 |
| original_firstorder_Variance | 6,70E-03 | 1 |
| lbp.3D.m1_firstorder_10Percentile | 8,00E-01 | 1 |
| lbp.3D.m1_firstorder_ExcessKurtosis | 2,50E-02 | 1 |
| lbp.3D.m1_firstorder_Minimum | 1,10E-01 | 1 |
| lbp.3D.m1_firstorder_Skewness | 1,30E-02 | 1 |
| lbp.3D.m2_firstorder_10Percentile | 9,00E-01 | 1 |
| lbp.3D.m2_firstorder_Minimum | 1,00E-01 | 1 |
| lbp.3D.m2_firstorder_Skewness | 4,90E-01 | 1 |
| lbp.3D.k_firstorder_90Percentile | 9,80E-03 | 1 |
| lbp.3D.k_firstorder_InterquartileRange | 9,60E-02 | 1 |
| lbp.3D.k_firstorder_MeanAbsoluteDeviation | 4,80E-02 | 1 |
| lbp.3D.k_firstorder_Minimum | 9,10E-01 | 1 |
| lbp.3D.k_firstorder_RobustMeanAbsoluteDeviation | 1,30E-01 | 1 |
| lbp.3D.k_firstorder_Variance | 1,90E-02 | 1 |
| original_glcm_ClusterShade | 1,10E-02 | 1 |
| original_glcm_Contrast | 7,10E-01 | 1 |
| original_glcm_DifferenceAverage | 5,80E-01 | 1 |
| original_glcm_DifferenceEntropy | 8,30E-02 | 1 |
| original_glcm_DifferenceVariance | 8,30E-01 | 1 |
| original_glcm_Idm | 4,00E-01 | 1 |
| original_glcm_MCC | 3,40E-01 | 1 |
| original_glcm_Id | 4,10E-01 | 1 |
| original_glcm_InverseVariance | 4,40E-01 | 1 |
| original_glcm_SumSquares | 2,30E-02 | 1 |
| original_glrlm_LongRunEmphasis | 9,50E-02 | 1 |
| original_glrlm_RunLengthNonUniformityNormalized | 2,10E-02 | 1 |
| original_glrlm_RunPercentage | 2,70E-02 | 1 |
| original_glrlm_RunVariance | 1,20E-01 | 1 |
| original_glrlm_ShortRunEmphasis | 2,50E-02 | 1 |
| original_glszm_LargeAreaEmphasis | 1,60E-02 | 1 |
| original_glszm_SizeZoneNonUniformityNormalized | 5,20E-01 | 1 |
| original_glszm_SmallAreaEmphasis | 7,20E-01 | 1 |
| original_glszm_ZonePercentage | 7,70E-02 | 1 |
| original_glszm_ZoneVariance | 7,90E-03 | 1 |
| original_ngtdm_Busyness | 7,80E-01 | 1 |
| original_ngtdm_Contrast | 2,60E-02 | 1 |
| original_ngtdm_Strength | 3,20E-01 | 1 |
| original_gldm_DependenceNonUniformityNormalized | 2,90E-02 | 1 |
| original_gldm_DependenceVariance | 2,00E-02 | 1 |
| original_gldm_LargeDependenceEmphasis | 4,40E-02 | 1 |
| original_gldm_SmallDependenceEmphasis | 1,30E-01 | 1 |

## SUPPLEMENTAL TABLE 5

**Selected features and importance ranking of radiomic prediction models.**

| **Feature** | **Feature coefficient** |
| --- | --- |
| *Radiomics-shape* | |
| original_shape_SurfaceVolumeRatio | -1.7764 |
| original_shape_Elongation | 0.6833 |
| Radiomics-intensity | |
| original_firstorder_90Percentile | 0.0077 |
| original_firstorder_RootMeanSquared | 0.0056 |
| *Radiomics-LBP* | |
| lbp.3D.k_firstorder_10Percentile | 7.463 |
| lbp.3D.k_firstorder_InterquartileRange | -2.9638 |
| lbp.3D.m2_firstorder_RobustMeanAbsoluteDeviation | 1.2288 |
| lbp.3D.m2_firstorder_Uniformity | -1.2241 |
| lbp.3D.k_firstorder_Skewness | 0.4799 |
| lbp.3D.m2_firstorder_ExcessKurtosis | -0.1825 |
| lbp.3D.k_firstorder_Energy | 5E-04 |
| *Radiomics-texture* | |
| original_glrlm_ShortRunEmphasis | -2.7036 |
| original_glcm_Imc2 | -2.1679 |
| original_glcm_Correlation | 1.2805 |
| original_glcm_JointAverage | 0.0775 |
| *Radiomics-combined* | |
| lbp.3D.k_firstorder_10Percentile | 5.9861 |
| lbp.3D.k_firstorder_InterquartileRange | -1.7338 |
| original_shape_SurfaceVolumeRatio | -0.9206 |
| lbp.3D.m2_firstorder_RobustMeanAbsoluteDeviation | 0.8037 |
| lbp.3D.m2_firstorder_ExcessKurtosis | -0.2954 |
| original_glcm_Correlation | 0.1766 |
| original_shape_Elongation | 0.1324 |
| lbp.3D.k_firstorder_Skewness | 0.0455 |
| original_glcm_JointAverage | 0.0283 |
| original_firstorder_RootMeanSquared | 0.0098 |
| original_firstorder_90Percentile | 0.0038 |

## SUPPLEMENTAL TABLE 6

**Area under the receiver operator characteristic curve (AUC) values of the prediction models for lymph nodes metastases without ComBat correction.**

Four radiomic models using different input features were compared with conventional measures. The respective area under the curve (AUC) values and 95% confidence intervals (95% CI) are depicted.

| **Model** | **Training cohort**  **n=87 LN** | **Testing cohort**  **n=66 LN** |
| --- | --- | --- |
|  | **AUC (95% CI)** | **AUC (95% CI)** |
| ***LN short diameter*** | 0.76 (0.65-0.86) | 0.84 (0.74-0.94) |
| ***LN Volume*** | 0.74 (0.62-0.84) | 0.79 (0.65-0.89) |
| ***Expert rating*** | 0.65 (0.59-0.70) | 0.67 (0.61-0.74) |
| ***Radiomics-texture*** | 0.82 (0.72-0.91) | 0.77 (0.63-0.89) |
| ***Radiomics-shape*** | 0.77 (0.66-0.86) | 0.83 (0.69-0.93) |
| ***Radiomics-intensity*** | 0.77 (0.65-0.87) | 0.74 (0.58-0.88) |
| ***Radiomics-LBP*** | 0.87 (0.78-0.94) | 0.91 (0.80-1.00) |
| ***Radiomics-combined*** | 0.89 (0.82-0.95) | 0.94 (0.87-0.98) |

## SUPPLEMENTAL TABLE 7

**Univariate and multivariate analysis for histology confirmed lymph node recurrence without ComBat Harmonization.**

Univariate and multivariate logistic regression of the linear predictors of the conventional parameters and the best performing radiomics model trained on radiomic features without ComBat harmonization (*Radiomics-combined*).

abbreviations: LN: lymph node. *significant result.

| **Clinical Variables** | **Univariate analysis** | | | **Multivariate analysis** | | |
| --- | --- | --- | --- | --- | --- | --- |
|  | **Odds ratio** | **95% CI** | **p value** | **Odds ratio** | **95% CI** | **p value** |
| *Expert rating* | 2.7 | 0-Inf | 1 | - | - | - |
| *LN short diameter* | 5.6 | 2-25 | 0.026***** | 2.34 | 0.59-15.2 | 0.34 |
| *LN Volume* | 9.8 | 2.1-8.6 | 0.08 | - | - | - |
| *Radiomics-combined* | 12 | 3.7-62 | 0.0018***** | 8.5 | 2.5-47.9 | 0.0036* |

## SUPPLEMENTAL TABLE 8

**Classification metrics of all tested models without ComBat Harmonization.**

Four radiomic models using different input features were compared with conventional measures. The respective performance metrics (Matthews correlation coefficient (MCC), balanced accuracy, sensitivity, specificity, positive predictive value (PPV), and negative predictive value (NPV)) determined on the test set are reported.

| **Model** | **MCC** | **Balanced Accuracy** | **Sensitivity** | **Specificity** | **PPV** | **NPV** |
| --- | --- | --- | --- | --- | --- | --- |
| ***LN short diameter*** | 0.32 | 0.67 | 0.35 | 1.00 | 1.00 | 0.29 |
| ***LN Volume*** | 0.40 | 0.74 | 0.71 | 0.77 | 0.92 | 0.42 |
| ***Expert rating*** | 0.32 | 0.67 | 0.35 | 1.00 | 1.00 | 0.29 |
| ***Radiomics-texture*** | 0.29 | 0.65 | 0.84 | 0.46 | 0.85 | 0.43 |
| ***Radiomics-shape*** | 0.37 | 0.73 | 0.61 | 0.85 | 0.94 | 0.37 |
| ***Radiomics-intensity*** | 0.25 | 0.54 | 1.00 | 0.08 | 0.80 | 1.00 |
| ***Radiomics-LBP*** | 0.80 | 0.87 | 0.98 | 0.77 | 0.94 | 0.91 |
| ***Radiomics-combined*** | 0.64 | 0.73 | 1.00 | 0.46 | 0.88 | 1.00 |

**SUPPLEMENTAL TABLE 9**

**Radiomics quality score of the study according to Lambin, P** ***et al.*** (3).

|  | **Criteria** | **Maximum Points** | **Study Score** |
| --- | --- | --- | --- |
| 1 | Image protocol quality | +1 (if protocols are well-documented)  +1 (if public protocol is used) | 2 |
| 2 | Multiple segmentations | +1 | 1 |
| 3 | Phantom study on all scanners | +1 | 0 |
| 4 | Imaging at multiple time points | +1 | 0 |
| 5 | Feature reduction or adjustment for multiple testing | 3 (if neither measure is implemented)  +3 (if either measure is implemented) | 3 |
| 6 | Multivariable analysis with non radiomic feature | +1 | 0 |
| 7 | Detect and discuss biological correlates | +1 | 0 |
| 8 | Cut-off analyses | +1 | 1 |
| 9 | Discrimination statistics | +1 (if a discrimination statistic and its statistical significance are reported)  +1 (if also an resampling method technique is applied) | 2 |
| 10 | Calibration statistics | +1 (if a calibration statistic and its statistical significance are reported)  +1 (if also an resampling method technique is applied) | 2 |
| 11 | Prospective study registered in a trial database | +7 (for prospective validation of a radiomics signature in an appropriate trial) | 0 |
| 12 | Validation | 5 (if validation is missing)  +2 (if validation is based on a dataset from the same institute)  +3 (if validation is based on a dataset from another institute)  +4 (if validation is based on two datasets from two distinct institutes)  +4 (if the study validates a previously published signature) +5 (if validation is based on three or more datasets from distinct institutes) *  Datasets should be of comparable size and should have at least 10 events per model feature. | 3 |
| 13 | Comparison to ‘gold standard’ | +2 | 2 |
| 14 | Potential clinical utility | +2 | 2 |
| 15 | Cost-effectiveness analysis | +1 | 0 |
| 16 | Open science and data | +1 (if scans are open source)  +1 (if region of interest segmentations are open source)  +1 (if code is open source)  +1 (if radiomics features are calculated on a set of representative ROIs and the calculated features + representative ROIs are open source) | 0 |
| **Total Score** | | **36 Points** | **19 Points (53%)** |

# SUPPLEMENTAL FIGURES

## SUPPLEMENTAL FIGURE 1

**Patients Workflow**


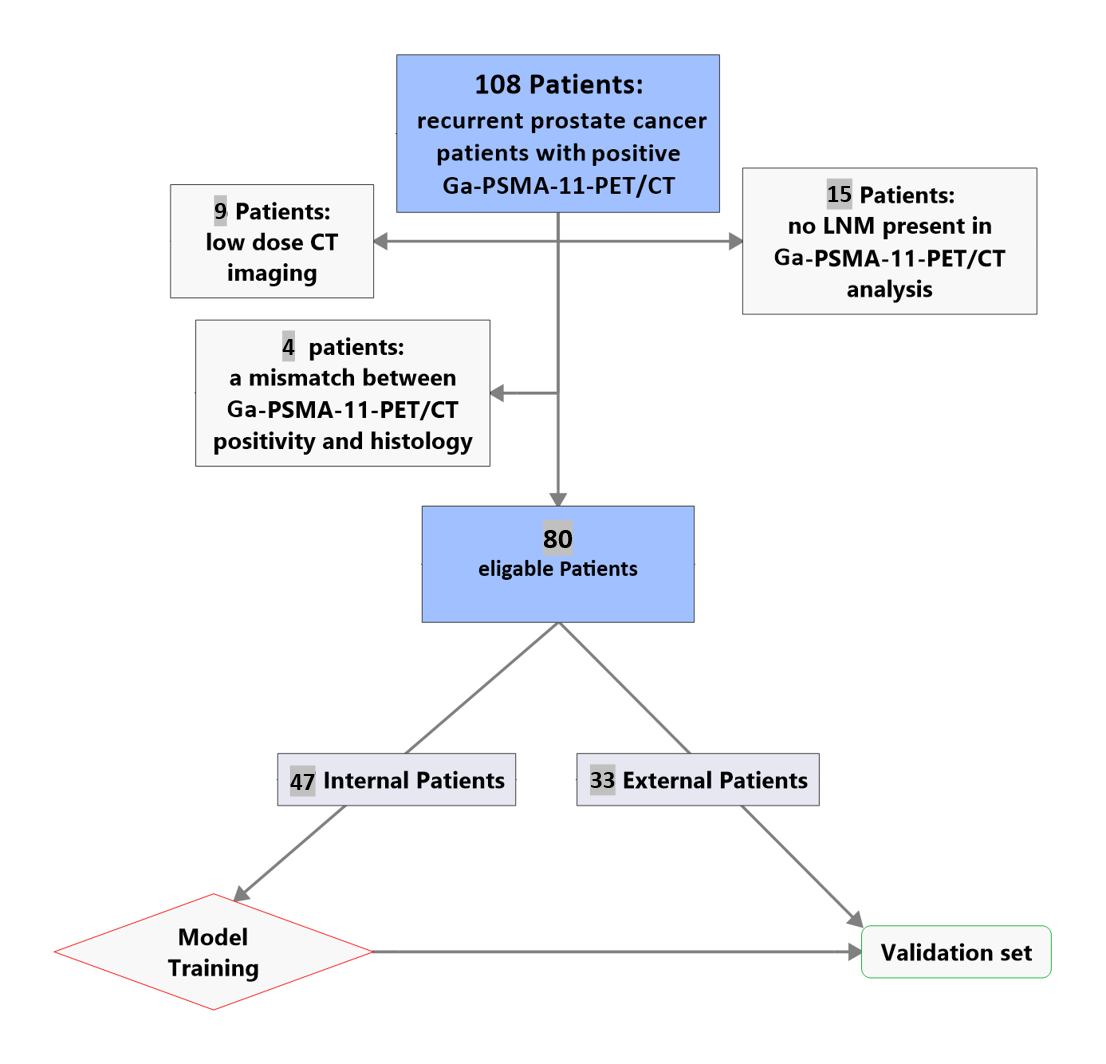


## SUPPLEMENTAL FIGURE 2

**Lymph node short diameter distribution in the training and test set.**

**
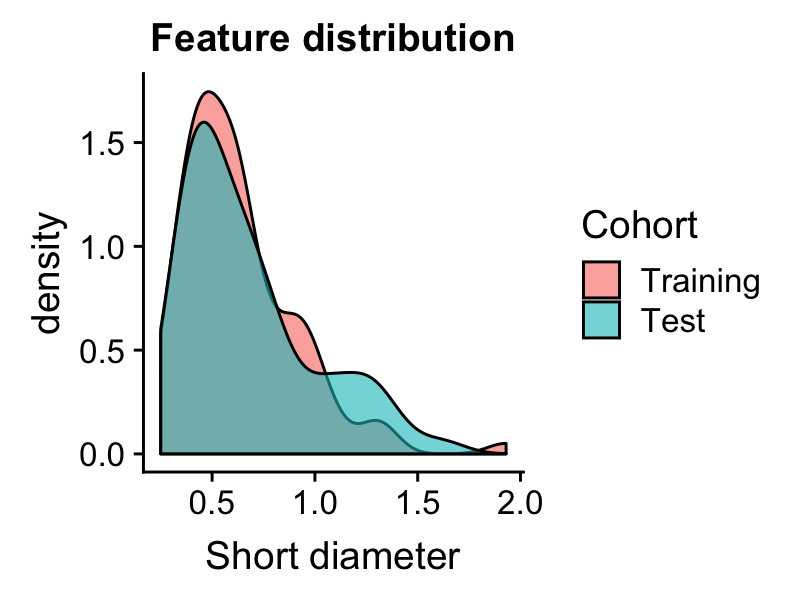
**

## SUPPLEMENTAL FIGURE 3

**Spearman’s rank correlation coefficient of texture features correlated to LN volume.**


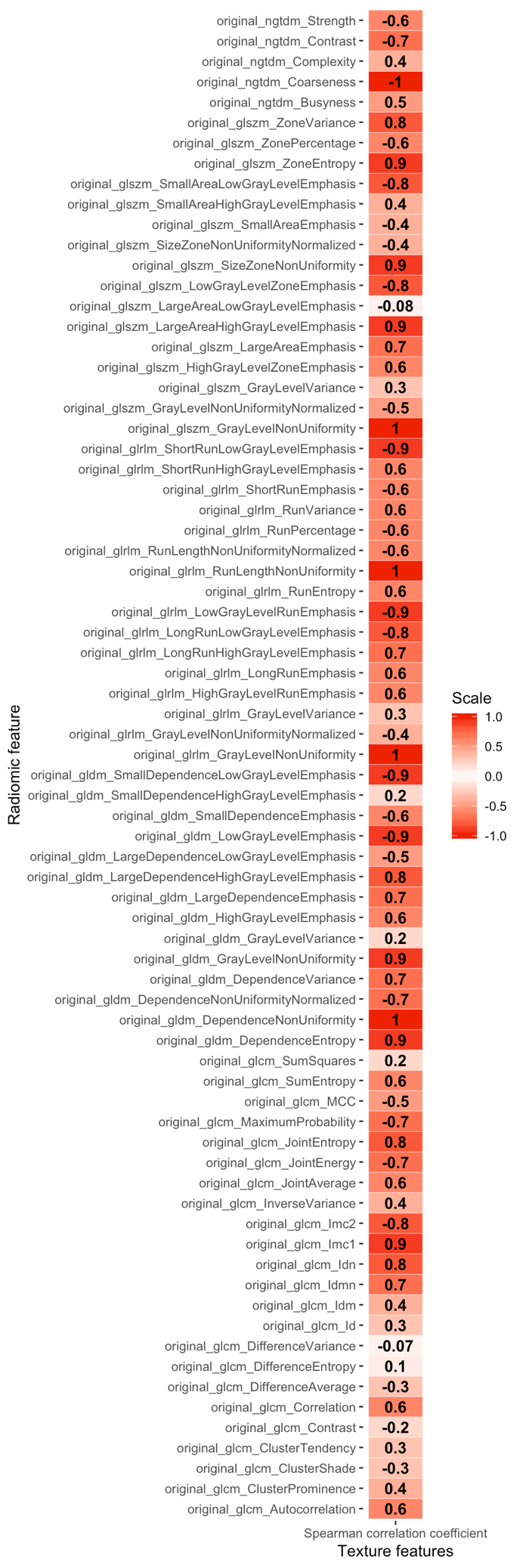


## SUPPLEMENTAL FIGURE 4

**Spearman’s rank correlation coefficient of LBP features correlated to LN volume.**


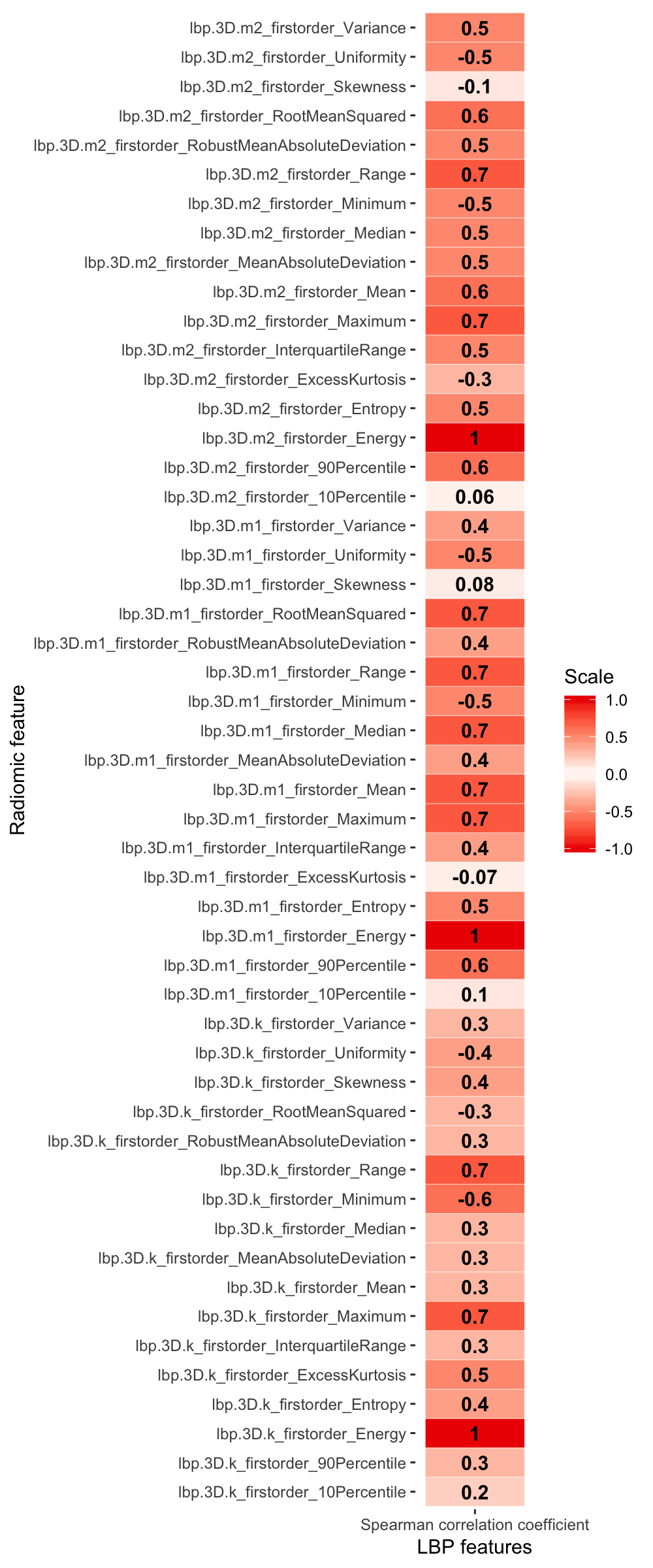


## SUPPLEMENTAL FIGURE 5

**Spearman’s rank correlation coefficient of shape features correlated to LN volume.**


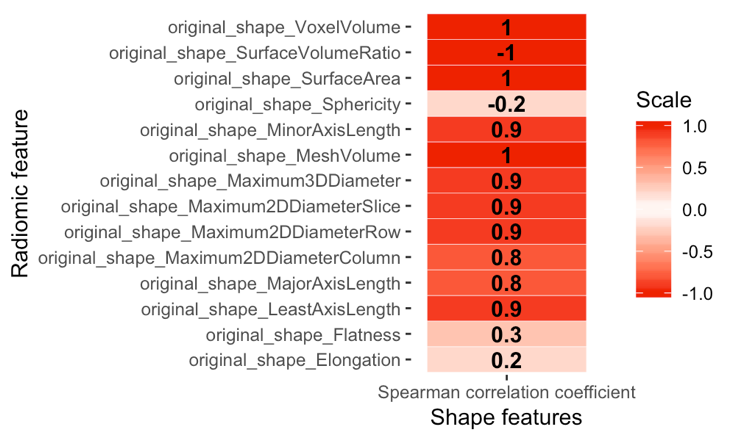


## SUPPLEMENTAL FIGURE 6

**Spearman’s rank correlation coefficient of intensity features correlated to LN volume.**


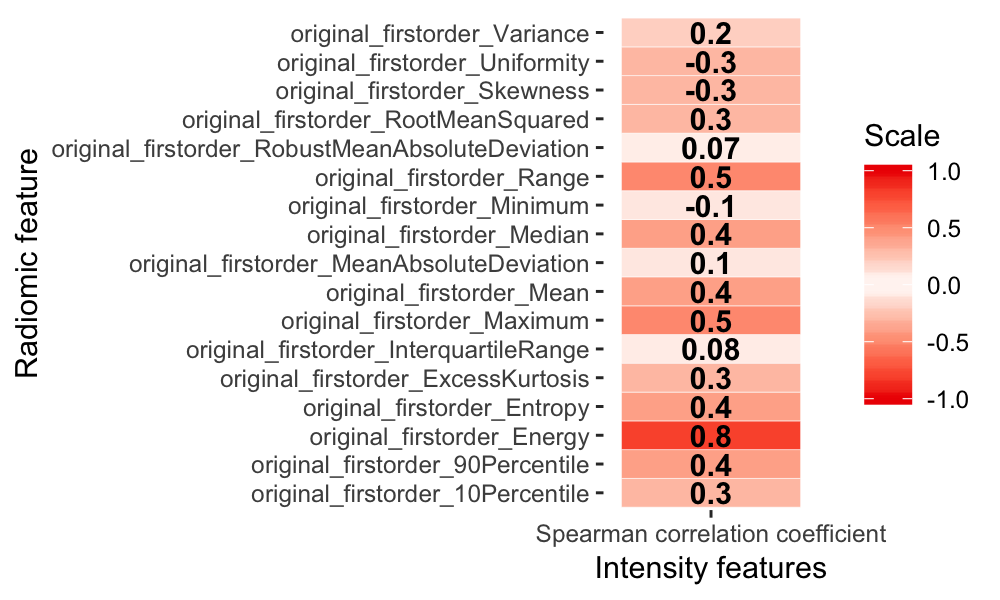


## SUPPLEMENTAL FIGURE 7

**Calibration curves of the developed prediction models for lymph node metastases status.**

| **Testing Training** |
| --- |

**
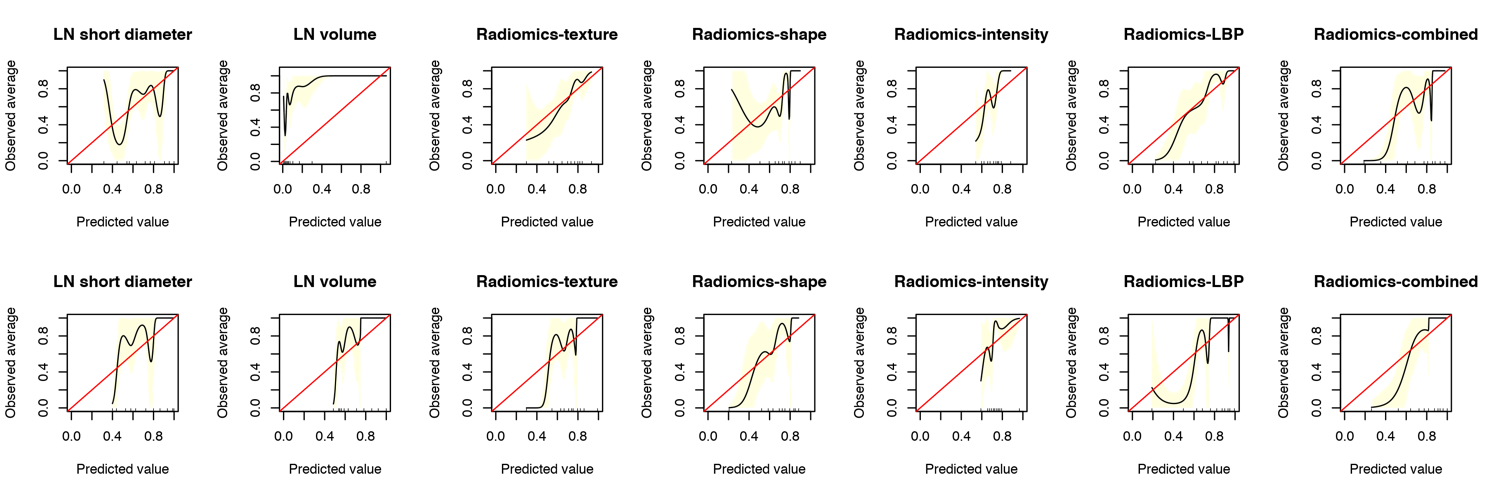
**

Calibration curves for the training set and validation set are depicted for LNM-status.

## SUPPLEMENTAL FIGURE 8

**Distribution of lymph node volume, short diameter and the Radiomics-combined score between histologically positive and negative lymph nodes in the test set.**

**
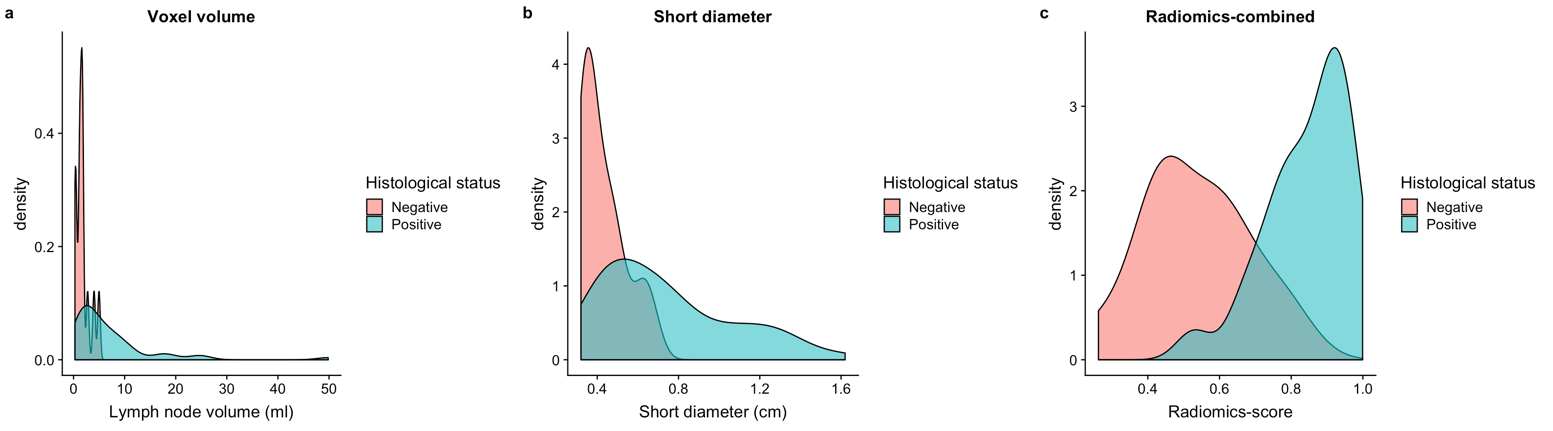
**

# References

1. Zwanenburg A, Leger S, Vallières M, Löck S. Image biomarker standardisation initiative. *ArXiv* [Internet] (2016);1612.07003. Available from: http://arxiv.org/abs/1612.07003

2. van Griethuysen JJM, Fedorov A, Parmar C, Hosny A, Aucoin N, Narayan V, et al. Computational Radiomics System to Decode the Radiographic Phenotype. *Cancer Res* [Internet] (2017);77(21):e104–7. Available from: http://cancerres.aacrjournals.org/lookup/doi/10.1158/0008-5472.CAN-17-0339

3. Lambin P, Leijenaar RTH, Deist TM, Peerlings J, De Jong EEC, Van Timmeren J, et al. Radiomics: The bridge between medical imaging and personalized medicine. *Nat Rev Clin Oncol* [Internet] (2017);14(12):749–62. Available from: http://dx.doi.org/10.1038/nrclinonc.2017.141
